# Anticoagulation management in elderly patients with proximal femur fractures – overview of current concepts

**DOI:** 10.1515/iss-2023-0030

**Published:** 2023-12-05

**Authors:** Yasmin Youssef, Anna K. I. M. Dietrich, Annika Hättich

**Affiliations:** Department of Orthopaedics, Trauma and Reconstructive Surgery, University Hospital Leipzig, 04103 Leipzig, Germany; Department of Trauma Surgery, Hannover Medical School, Hannover, Germany; Department of Trauma and Orthopaedic Surgery, University Hospital Hamburg Eppendorf, Hamburg, Germany

**Keywords:** proximal femur fracture, anticoagulation, anticoagulation management, comorbidities, tranexamic acid

## Abstract

**Objectives:**

Proximal femur fractures (PFF) are common injuries in elderly patients and can have considerable effects on their quality of life, morbidity, and mortality. Due to pre-existing comorbidities, the prevalence of anticoagulated patients is increasing. The right timing for surgery and perioperative anticoagulation treatment remains controversial.

**Content:**

This overview aims to summarize current practices in the pre- and postoperative anticoagulation management and the recommended time to surgery in elderly patients with PFF.

**Summary and Outlook:**

Time to surgery for anticoagulated patients is often prolonged due to worries about serious perioperative bleeding and higher transfusion demands. But the delay of surgical PFF treatment increases the risk for perioperative complications like pulmonary embolism, pneumonia, deep vein thrombosis and urinary tract infections. Early surgery can be achieved with a consistent and interdisciplinary perioperative anticoagulation management. Antiplatelets do not have to be discontinued and surgery should be performed early without delay. For patients taking vitamin K antagonists (VKA) an INR less than 1.5 is recommended prior to surgery, which can be achieved by pausing VKA intake or by administering vitamin K, prothrombin complex concentrate (PCC) or fresh frozen plasma (FFP). For the treatment with direct oral anticoagulants (DOAC) a plasma drug level of under 50 pg/mL is considered safe for surgery. If the plasma level can not be determined, a gap of 24 h between the last DOAC dose and surgery is recommended. The systemic administration of tranexamic acid can reduce overall blood loss and transfusion rates in anticoagulated patients with PFF. Surgical treatment of PFF should be performed within 24 h, as delayed surgery increases the risk for perioperative complications. This also applies to anticoagulated patients, when clinically appropriate. International and interdisciplinary guidelines are necessary to ensure early and appropriate treatment of anticoagulated elderly patients with PFF.

## Introduction

Proximal femur fractures (PFF) are common injuries in elderly patients [1], [2], [3]. Higher average life expectancy and lifestyle changes in the elderly populations are predicted to lead to a continuous rise of PFF [2]. In Germany their incidence increased approximately by 24 % between 2009 and 2019 [4]. In 2050, the global incidence is estimated to be seven million [2].

PFF, can be categorized into femoral head and neck fractures, pertrochanteric and subtrochanteric fractures. The surgical treatment depends on the fracture morphology as well as individual patient factors (e.g., pre-fracture mobility, lifestyle etc.) [5]. Common procedures include total- and hemiarthroplasty, intramedullary nailing, triple screw fixation and dynamic hip screw fixation [5]. PFF can cause a significant perioperative blood loss, drop in haemoglobin and an increased need for transfusions preceding surgical treatment [6]. Risk factors for bleeding include the presence of comorbidities, intertrochanteric fractures, and a prolonged time to surgery [6].

Elderly patients with PFF often suffer from osteoporosis and are frail [7], meaning they have an impaired functional capacity and are more vulnerable to external stress factors. Up to 50 % of all patients with hip fractures have pre-existing nursing care needs [8]. Therefore PFF and their subsequent invasive surgical treatment pose a life changing experience for many elderly patients, which can have considerable effects on their quality of life, mortality, and morbidity. PFF in elderly patients are associated with higher risks for cardiovascular, pulmonary, thrombotic, infectious, and haemorrhagic complications [9]. During the first postoperative year mortality rates of up to 30 % are described [4].

Due to the high number of cardiovascular and cerebrovascular comorbidities in frail and elderly patients, many are under permanent anticoagulation. Studies suggest that up to 53.6 % of the patients are anticoagulated at the time of fracture [1, 3, 5]. In the past, the perioperative anticoagulation management in elderly patients with PFF was often approached with caution, as it was though that extended preoperative disruption was necessary to prevent complications like considerable bleeding, higher transfusion demand and the development of (post-operative) haematoma [10]. In consequence, anticoagulated patients tend to have delayed times to surgery [1]. However, current literature suggests that prolonged time to surgery correlates with higher morbidity and mortality rates [2, 10, 11]. A delay of more than 24 and 48 h between patient admission and surgery is associated with an increased risk of perioperative complications like wound-, urinary-tract- and respiratory-infections, decubitus, lung oedema, pneumonia and pulmonary embolism, deep vein thrombosis and impaired mobility [1, 2, 11, 12].

The purpose of this review is to give a concise overview of current practices of pre- and postoperative management of antiplatelets, VKA and DOAC in elderly patients with PFF as well as the optimal time for surgery and the evidence for the admission of tranexamic acid.

## Methods

**Figure 1:**
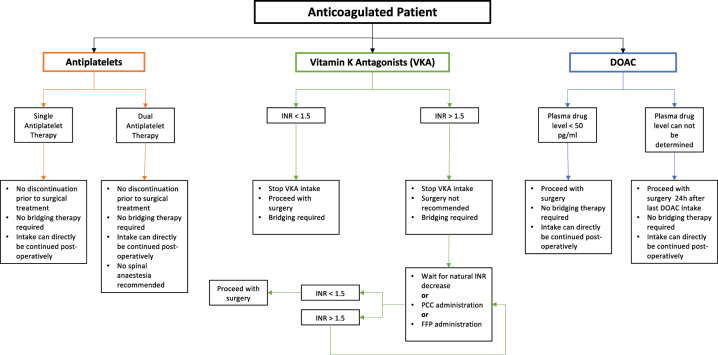
Algorithm for the management of anti coagulated patients.

A scoping literature research of all studies published on the topic since 2020 has been performed using PubMed. All published papers were evaluated by two of the authors independently. The information collected by the individual authors was sorted by substance class. Finally, the retrieved information, including the perioperative management of the substances as well as the recommended time-to surgery was discussed by all authors and summarized comprehensively (Table 1, Figure 1).

**Table 1:** Pharmacological and clinical information of the most frequently used anticoagulants.

	Common substances	Half-life (h)	Mode of action	Preoperative laboratory	Preoperative management	Intraoperative management	Postoperative management
Antiplatelets	ASS	4–20	Irreversible inhibition of COX-1	Not required	No discontinuation required	If necessary platelet concentrates	Continue according to indication
	Clopidogrel Prasurgrel Ticagrelor	7–8	Irreversible inhibition of P2Y12				
VKA	Warfarin	40	Inhibition of vitamin K dependent γ-carboxylation of the clotting factors II, VII, IX and X	INR	Discontinuation with bridging required; INR<1.5 necessary for surgery; if indicated vitamin K or PCC administration	If necessary PCC	INR according to indication; if necessary, bridging with LMW heparin
	Phenprocoumon	140					
DOAC	Dabigatran	12–14	Direct inhibition of factor II	Not required; where applicable plasma level	Wait at least 24 h after last intake or plasma level <50 pg/mL	If necessary Idarucizumab	Continue according to indication; bridging with LMW heparin if continuation is not possible
	Rivaroxaban	9–13	Direct inhibition of factor X			If necessary PCC	
	Apixaban	8–15					
	Edoxaban	9–11					

## Results and discussion

### Antiplatelets

The most used antiplatelets are aspirin (ASS), clopidogrel, prasugrel and ticagrelor. Further agents are available but are not considered in the following. Their common effect is to prevent platelet aggregation and clot formation. ASS irreversibly inhibits cyclooxygenase-1 (COX-1), leading to a reduced production of thromboxane A2. The more potent substances clopidogrel, ticagrelor and prasugrel bind to the platelet receptor P2Y_12_ and thereby inhibit ADP-mediated clotting signal pathways [2]. Except for ticagrelor the effects are irreversible. Due to the irreversible inhibitory action of antiplatelets their effect lasts for approximately seven days, corresponding to the mean platelet lifetime [10].

Common indications for antiplatelet therapy are prevention and treatment of ischemic heart disease, stroke, peripheral arterial occlusive disease and other prothrombotic conditions. Dual antiplatelet therapy defines the combination of two antiplatelets with two different effects. Typically, ASS and clopidogrel are used, e.g., as prophylaxis for thrombosis after stent implantation. Spinal anaesthesia is proven safe under ASS. In case of P2Y_12_ inhibitor intake five to seven days are recommended between withdrawal and spinal anaesthesia to avoid spinal haematoma. In patients with dual antiplatelet therapy spinal anaesthesia is contraindicated [2, 12].

Prior to surgical PFF treatment antiplatelets do not have to be discontinued. Dual antiplatelet treatment has also been proven safe [2]. Surgery should proceed as early as possible without delay. Currently, no guideline exists that defines the optimal time to surgery for patients with PFF on antiplatelet drugs [10]. However current literature suggests waiving withdrawal in favour of rapid surgical treatment and prevention of thrombotic events [2, 5, 12]. Especially the risk for stroke and myocardial infarction increases during surgery and is to be averted [11].

Yang et al. could show that there are no significant differences between patients on antiplatelets and non-anticoagulated patients concerning the drop in haemoglobin, the units of blood transfusions, the one-year-mortality, the length of hospital stay, the occurrence of cerebrovascular events, deep vein thrombosis, pulmonary embolism, major bleeding, and wound-related complications following hip fracture surgery [10].

The effect of platelet inhibitors can be reversed in case of severe bleeding by transfusion of platelets [2, 11]. However it must be noted that they are ineffective in patients taking ticagrelor and prasugrel [2].

### Vitamin K antagonists

The substance class of vitamin K antagonists includes the agents warfarin, acenocoumarol and phenprocoumon. VKA inhibit the vitamin K dependent γ-carboxylation of the clotting factors II, VII, IX and X and the antithrombotic proteins C and S in the liver. Typical indications for VKA among others are atrial fibrillation, thrombosis (e.g., deep vein thrombosis and pulmonary embolism), mechanical heart valves and other prothrombotic conditions [2]. The prevalence of warfarin medication in PFF patients is estimated to be between 5.0 and 10.3 %. The drug action can be monitored by the international normalised ratio (INR). Target range for most indications is an INR between 2.0 and 3.0 [2].

Surgical treatment of PFF is only recommended in patients with an INR less than 1.5. The INR can be decreased by pausing VKA intake and waiting for natural INR decrease. However, this can last up to five days. For this reason, this option is not considered acceptable with respect to the proven benefits of early surgery [2]. Still, it is estimated that only 20 % of warfarinised patients receive PFF surgery within the first 48 h after trauma due to a consistent high INR [2]. A faster INR correction can be achieved by oral or intravenous vitamin K substitution [2]. There is no consensus regarding the optimal dose, time frame and administration form of vitamin K. Intravenous vitamin K administration has been found to result in a decreased time to surgery, a low complication rate and shorter length of hospital stay [2]. Moreover, oral administration is not considered as effective compared to intravenous application [2].

Furthermore, the INR can be decreased by the administration of prothrombin complex concentrate (PCC) or fresh frozen plasma (FFP) before surgery. PCC contains a 25-times higher concentrations of inactive clotting factors (II, VII, IX, X) and protein C and S, compared to average natural concentrations. Co-administration of 5 mg vitamin K (p.o.) is advised because of an INR rebound 6 h after substitution [2]. FFP is considered less effective compared to PCC in restoring normal INR with the added risk of volume overload and thromboembolic events [2]. The current literature does not support its use in VKA reversal for PFF surgery [2]. Ultimately, it is important that the benefit of reversal of VKA is balanced with the increased risk of thrombotic events [3]. Therefore, bridging with treatment-dose subcutaneous low-molecular-weight heparin (LMH) or intravenous unfractionated heparin should be considered in patients with mechanical heart valves and atrial fibrillation with recent history of stroke, deep vein thrombosis or pulmonary embolism [5].

There are different opinions about restarting VKA after surgery. Papachristos and Giannoudis recommended restarting warfarin between 24 and 36 h postoperatively. This time frame does not prolong the length of hospital stay and seems to be safe. Bridging with LMH is a standard procedure after VKA discontinuation [2].

Compared to non-anticoagulated patients, patients with PFF under VKA therapy do not only have longer intervals to surgery (50 % vs. 25 % without surgical treatment 24 h after admission), but also have longer hospital stays (17.1 days vs. 15.1 days) and have an approximately 1.5-times higher risk for revision surgery. 

### Direct oral anticoagulants

Direct oral anticoagulants (DOAC) are a group of anticoagulants that inhibit a specific clotting factor and are administered orally. Among others this group includes dabigatran (direct thrombin inhibitor), apixaban, edoxaban and rivaroxaban (direct factor Xa inhibitors).

A plasma drug level of under 50 pg/mL is considered safe for surgery in patients taking factor Xa inhibitors [5, 11, 12]. Plasma levels higher than 100 pg/mL can lead to severe bleeding during surgery [11]. If there is no possibility to determine the plasma level, a gap of 24 h between the last DOAC dose and surgery should be considered [3, 5]. However it must be noted that DOAC are eliminated renally and therefore accumulate in patients with chronic or acute kidney failure. This increases the risk of perioperative bleeding and thromboembolism [11, 13]. In patients with end-stage renal insufficiency apixaban, edoxaban and rivaroxaban seem more suitable than dabigatran [11].

In case of the potential threat of bleeding or the wish for early/immediate surgery, reversal agents can be applied. The reversal agent for dabigatran is Idarucizumab. Andexanet alfa antagonises factor Xa inhibitors, but approval only exists for apixaban and rivaroxaban. In case those reversal agents are not available, alternatives include PCC with activated or inactivated clotting factors [3, 5, 11].

A recent study which presented data from the multicentric German trauma register, investigated the effect of DOAC in the treatment of geriatric hip fracture patients. The study showed that 11.0 % of the included patients were under DOAC therapy compared to 9.2 % under VKA therapy [1]. There was no significant difference between patients taking DOAC and VKA with regards to the occurrence of perioperative complications, type of anaesthesia and mortality rate.

The time to surgery was significantly longer in patients taking DOAC or VKA, compared to the control group without any anticoagulation [1]. While 75 % of the control group were surgically treated within the first 24 h, only 46 % of the patients with DOAC and 50 % of the patients under VKA therapy received early surgical treatment (before 24 h) [1]. The presented hazard ratios showed both DOAC and VKA intake reduced the probability for early surgery by a factor of 0.6 regardless of age and ASA classification [1]. Schuetze et al. who examined the impact of oral anticoagulation on proximal femur fractures treated within 24 h showed that patients under DOAC therapy had a 3.4-fold increased risk of intraoperative blood transfusions [14].

In contrast to VKA, no bridging with LMH must be performed preoperatively because of the short half-life of DOAC. They can be directly reinserted postoperatively [11].

### Tranexamic acid

Tranexamic acid is a synthetic derivative of the amino acid lysine and acts as an antifibrinolytic agent. By inhibiting plasmin formation tranexamic acid slows down fibrinolysis and increases blood-clot stability. In various surgical disciplines tranexamic acid is used prophylactically to decrease intraoperative bleeding and transfusion requirements without increasing the risk for thromboembolic complications [2, 15, 16]. Across all surgical specialties the most frequent dose of tranexamic acid is 15 mg/kg [15]. Contraindications for its use include pulmonary diseases (e.g., pulmonary hypertension), coagulopathies and thrombotic events like myocardial infarction, deep vein thrombosis, stroke, or pulmonary embolism in patient history. However there remains a paucity of research on the prophylactic use of tranexamic acid in different surgical subareas and universal standards are necessary.

It has been shown that the systemic use of tranexamic acid can reduce overall blood loss and transfusion rates in anticoagulated patients with PFF [5, 12, 16]. However, evidence about the appropriate timing, administration form and dosage of tranexamic acid in those patients is still missing [5, 12]. Especially its application in patients under DOAC therapy is not fully studied and needs further research.

A prospective randomised controlled trial by Ekinci et al. examined the effect of the systemic application of tranexamic acid in elderly patients with intertrochanteric femur fractures who were treated by proximal femur nailing. The study group (n=51) received 15 mg/kg of tranexamic acid preoperatively whereas the control group (n=51) was given only isotonic saline. The groups did not differ in terms of sociodemographic characteristics as well as preoperative haemoglobin and haematocrit values [17]. In this study it could be shown that the systemic application of tranexamic acid significantly reduces total blood loss, hidden blood loss and the need for blood transfusions [17]. The mean total blood loss in the tranexamic acid group was 684.6±370.1 mL while it was 971.2±505.3 mL in the control group (p=0.002) [17]. The transfusion rate was 8 % in the tranexamic acid group and 23.5 % in the control group (p=0.003). There was no significant difference found for the amount of intraoperative blood loss and the risk of deep vein thrombosis and thromboembolic events between the groups [17].

A recent study by Li et al. prospectively compared intravenous vs. oral application of tranexamic acid in patients with intertrochanteric femur fractures, treated with intramedullary nailing. The blood transfusion rate and the total blood loss were higher in the control group than in the group receiving oral or intravenous tranexamic acid. There was no significant difference between the oral and the intravenous group found for those parameters [18]. In addition, there were no differences in the rate of thromboembolic events between the oral and intravenous group [18]. These findings suggest that both oral and intravenous tranexamic acid seem to be safe.

Fenwick et al. compared 274 patients with PFF and tranexamic acid administration to 1,205 patients without tranexamic acid application. The study demonstrated that the postoperative haemoglobin drop was less when tranexamic acid was administered (1 g tranexamic acid: systemic, topic or combined application) and that transfusion rates were lowered significantly by 17.1 % [4]. When comparing the modes of administration, it was found out that the lowest blood loss could be achieved when tranexamic acid was only applied locally (895.86 mL; range: 453.5–1,176.2 mL; SD: 237.78 mL, p<0.000). In case of combined intravenous and local application the average blood loss was 1,139.55 mL; (range: 73.4–4,107 mL; SD: 810.18 mL). These results might however be biased due to the large inhomogeneity between the patient groups [4].

### Time to surgery

Current data suggest the timing of surgery in the treatment of PFF significantly influences the clinical outcome. Surgery within 48 h was associated with lower mortality rates and perioperative complications and was recommended as a practical standard in the past [9]. Today the updated guideline of the American Academy of Orthopaedic Surgeons recommends that hip surgery should be performed within 24 h [3]. Further delay of surgery increases the risk for perioperative complications like pulmonary embolism, pneumonia, deep vein thrombosis and urinary tract infection [2, 3, 5].

Despite the mentioned complications, anticoagulated patients still have longer times to surgery. Tran et al. showed that the time to surgery was 40.0 h in anticoagulated patients, while it was 26.2 h in non-anticoagulated patients [19]. Patients taking DOAC had the longest time to surgery with 66.9 h [19]. These results were supported by Aigner et al. [1]. At the same time, time to surgery of at least 48 h was shown to increase blood loss independent of anticoagulation [20]. Similarly Stacy et al. stated anticoagulation treatment had no effect on subsequent haemoglobin concentrations in patients with PFF [6]. Due to these studies, an early surgical treatment within the first 24 h after diagnosing a PFF is recommended to avoid perioperative complications and a prolonged stay in hospital.

## Conclusions

The treatment of PFF in anticoagulated elderly patients remains a challenge. Anticoagulated patients have an increased risk of delayed surgical treatment because of persisting concerns about bleeding complications and missing guidelines for perioperative anticoagulation management and time to surgery. Delayed surgery was shown to be associated with higher complication and morbidity rates. Current research suggests that a 24 h cut-off for the surgical treatment of PFF should also be adhered to in anticoagulated patients when clinically appropriate and reasonable. Substance class of anticoagulation as well as the last intake and renal function should be considered in the decision algorithm. Standardised interdisciplinary (orthopaedic surgeons, haematologists, and anaesthesiologists etc.) guidelines and algorithms are indispensable to ensure optimal treatment of those patients.
